# AMMOS: Automated Molecular Mechanics Optimization tool for *in silico *Screening

**DOI:** 10.1186/1471-2105-9-438

**Published:** 2008-10-16

**Authors:** Tania Pencheva, David Lagorce, Ilza Pajeva, Bruno O Villoutreix, Maria A Miteva

**Affiliations:** 1 INSERM U648, Bioinformatics-MTI University Paris Diderot, 5 rue Marie-Andrée Lagroua, 75205 Paris Cedex 13, France; 2 Centre of Biomedical Engineering "Prof. Ivan Daskalov", Bulgarian Academy of Sciences, 105, Akad. Georgi Bonchev Str., 1113 Sofia, Bulgaria

## Abstract

**Background:**

Virtual or *in silico *ligand screening combined with other computational methods is one of the most promising methods to search for new lead compounds, thereby greatly assisting the drug discovery process. Despite considerable progresses made in virtual screening methodologies, available computer programs do not easily address problems such as: structural optimization of compounds in a screening library, receptor flexibility/induced-fit, and accurate prediction of protein-ligand interactions. It has been shown that structural optimization of chemical compounds and that post-docking optimization in multi-step structure-based virtual screening approaches help to further improve the overall efficiency of the methods. To address some of these points, we developed the program AMMOS for refining both, the 3D structures of the small molecules present in chemical libraries and the predicted receptor-ligand complexes through allowing partial to full atom flexibility through molecular mechanics optimization.

**Results:**

The program AMMOS carries out an automatic procedure that allows for the structural refinement of compound collections and energy minimization of protein-ligand complexes using the open source program AMMP. The performance of our package was evaluated by comparing the structures of small chemical entities minimized by AMMOS with those minimized with the Tripos and MMFF94s force fields. Next, AMMOS was used for full flexible minimization of protein-ligands complexes obtained from a mutli-step virtual screening. Enrichment studies of the selected pre-docked complexes containing 60% of the initially added inhibitors were carried out with or without final AMMOS minimization on two protein targets having different binding pocket properties. AMMOS was able to improve the enrichment after the pre-docking stage with 40 to 60% of the initially added active compounds found in the top 3% to 5% of the entire compound collection.

**Conclusion:**

The open source AMMOS program can be helpful in a broad range of *in silico *drug design studies such as optimization of small molecules or energy minimization of pre-docked protein-ligand complexes. Our enrichment study suggests that AMMOS, designed to minimize a large number of ligands pre-docked in a protein target, can successfully be applied in a final post-processing step and that it can take into account some receptor flexibility within the binding site area.

## Background

Structure-based virtual ligand screening (SBVLS) allows to investigate thousands or millions of molecules against a biomolecular target [1,2], and as such it plays an increasingly important role in modern drug discovery programs. For example, numerous SBVLS methods employing docking and scoring have been developed to assist the discovery of hit compounds and their optimization to leads [3-5]. These methods orient and score small molecules in a protein-binding site, searching for shape and chemical complementarities. Many novel active compounds acting on key therapeutic targets have been found through combining SBVLS and in vitro screening experiments [5,6]. Despite the considerable progresses achieved these recent years, several problems are still present in most of the currently available SBVLS packages. Among the most critical is the flexibility of the receptors that frequently change their conformations upon ligand binding. Several methods have been developed to attempt to take into consideration receptor flexibility during docking/scoring [2,7-10], however, this is still very challenging because the number of conformations rises exponentially with the number of rotatable bonds and the full sampling of all possible conformations is not feasible for a large number of protein-ligand complexes.

Further the correct prediction of receptor-ligand binding energies [11,12] and accurate ranking of the compounds with respect to their estimated affinities to a target remains highly challenging. Thus it is still difficult to discriminate bioactive compounds from false positives [13,14] despite recent efforts to improve enrichment via, for instance, docking on different protein targets [15] or through optimized or new scoring functions [12,16,17]. In addition, and among the many players that are important in SBVLS computations, the quality of the screened chemical libraries has also been shown to be important in order to correctly predict the bound ligand-conformations and for ranking [18,19]. Within this context, further refinements and optimization of VLS docking-scoring methods are needed.

Recently it has been suggested that post-docking optimization, either after conventional docking-scoring procedures or after hierarchical VLS protocols [20-23] may help to further improve both, the docking pose and the scoring, and as such the overall efficiency of SBVLS experiments. Recent examples of docked poses and enrichment improvements after post-docking energy minimization support this view [19,24-27].

In the present study, we propose a new open source program, named AMMOS, which addresses some of the pre- and post-processing problems associated with SBVLS computations, through molecular mechanics (MM) modeling. AMMOS executes an automatic procedure for: (1) energy minimization of pre-docked protein-ligand complexes allowing partial or full atom flexibility from both, the ligand and the receptor sides and (2) structural optimization of chemical compounds present in the screening libraries prior to docking experiments. MM is currently a very reliable approach to model protein-receptor interactions in a physically realistic manner [26-28] since it can account for local flexibility adjustments from both, the protein and the ligand although conformational exploration is not possible if large conformational changes occur. It is indeed reasonable to apply such framework instead of more computer demanding simulations (for instance molecular dynamics) in large-scale applications involving the handling of thousands of compounds. In conventional MM studies, the bonded interactions include the bonds, bond angles and dihedral terms while the non-bonded interactions involve the van der Waals term represented by the Lennard-Jones (LJ) 6–12 potential, and electrostatic interactions, often treated by Coulombic potential computed between point charges centered on relevant atoms. AMMOS proposes relatively fast energy minimization by making use of the full-featured molecular mechanics program AMMP [28-31]. AMMP is available upon GNU license and has been recently implemented in the well-known OpenGL molecular modeling package VEGA [32]. In particular, VEGA implements AMMP for energy minimization with all the available optimization methods. However, to the best of our knowledge, VEGA can not minimize chemical libraries nor a large number of pre-docked protein-ligand complexes. AMMP has several advantages that make it relevant for the present applications such as: a fast multipole method for including all atoms in the calculation of long-range potentials and robust structural optimizers. AMMP has a flexible choice of several bonded and non-bonded potentials and permits analysis of individual energy terms. An additional advantage of AMMP is that it allows straightforward introduction of non-standard polymer linkages and non-standard amino-acid residues as well as manipulation of both small molecules and macromolecules including proteins, nucleic acids and other polymers. Furthermore, extensive benchmarking of AMMP has been performed highlighting its accuracy in term of energy minimization of proteins (i.e., the changes of atomic positions after minimization have been shown to be within the range of experimentally obtained variations in different crystal forms [33] and the calculated protein-ligand interaction energies have been successfully correlated with measured ligand binding affinities [34]). Up to now, AMMP has been successfully applied in numerous modeling studies of proteins and protein-ligand complexes [28,29,34,35] but it has not been used thus far for SBVLS computations as numerous implementations would be necessary to apply it automatically on thousands of small molecules, either present in a database or docked in a receptor binding site.

In this article we describe the development, implementation and evaluation of the AMMOS approach for automatic energy minimization of protein-ligand complexes or of small organic molecules. First energy minimization with AMMOS was validated by refinement of a large chemical library and by comparison of small molecule optimizations with two well established force fields available in the package SYBYL [36]. The efficiency of AMMOS for optimization of pre-docked protein-ligand complexes was then examined through performing calculations on two protein targets, namely estrogen receptor (ER) and neuraminidase (NA). These two proteins were selected because they are often used in SBVLS studies and because their binding pockets display rather different physicochemical properties and geometries. We used a multi-step SBVLS protocol to generate protein-ligand complexes. Our first VLS stage employed a search for satisfactory shape complementarity allowing rational reduction of the size of the initial chemical library. The pre-docked complexes subjected to AMMOS minimization were generated via the second step involving flexible-ligand docking. Finally we tested AMMOS as a final re-scoring engine on the selected pre-docked protein-ligand complexes for both, ER and NA. The obtained results are promising and suggest that AMMOS can be successfully applied as a post-processing tool to improve enrichment.

## Methods

### AMMP Molecular Mechanics

AMMOS makes use of the molecular simulation package AMMP [30], a program that can easily be embedded in other packages. AMMP allows to introduce standard or non-standard polymer linkages (ensured by the program PREAMMP included in the package AMMP), unusual ligands or non-standard residues, as well as to complete partial protein structures. AMMP incorporates a fast multipole algorithm for the efficient calculation of long-range forces thereby allowing evaluation of non-bonded terms without the use of a cutoff radius and increasing the speed, making calculations comparable to a standard treatment with a 8–10 Å radius cutoff [28]. The AMMP force field [28] is developed on the basis of the UFF potential set [37] and the AMBER partial charges [38]. The initial UFF set has been optimized for biological molecules [28,29] in order to improve the agreement with experimental geometry and spectral data. The first developed AMMP force field was the set *sp4 *[28]. Lately, Bagossi and co-authors [39] proposed the *Mo*dified *P*arameter *S*et for AMMP (MOPSA) with improved generation of partial charges for a wider range of compounds especially adapted for modeling of macromolecules, where the electrostatic parameters have been modified to achieve better correlation with experimental dipole moments. MOPSA parameter set has been merged with the AMMP parameter set *sp4 *to generate the new standard force field set *sp5 *[39].

We tested several minimization methods as implemented in AMMP. The steepest descent method [40] (first order) that uses the first derivatives of the energy function to find a local minimum, is available in AMMP, but this does not necessarily produce the fastest convergence. The Poliak-Ribeire Conjugate Gradient method (first order) [40], performing a search along conjugate directions, can produce generally faster convergence, is also implemented in AMMP. For the second-order minimization methods, the Broyden-Fletcher-Goldfarb-Shanno (BFGS) approach [41] that belongs to the Quasi-Newton methods [40], is available in AMMP. The non-derivative polytope simplex method [41], as well as a genetic algorithm (GA) [42] (evolutionary algorithms that perform directed random search to find the optimal solution in a complex multidimensional space) are also implemented in AMMP.

### Energy minimization protocols

#### Energy minimization of small organic molecules using AMMOS

We explored both AMMP force fields, *sp4 *and *sp5*, on a set of four small molecules structures generated by OMEGA 2.0 [43] (see Compound collections below). To speed-up ligand parameterization, partial charges on ligand atoms were assigned with the Gasteiger-Marsili method [44] using the OpenBabel package [45]. The maximum number of iterations was set to 5000. All calculations were performed with a convergence value set to 0.01 or 0.02 kcal.mol^-1^.Å^-1^, and no essential differences were observed. Thus, we chose 0.02 kcal.mol^-1^.Å^-1 ^as convergence criterion to reduce the computational time. The number of iterations required to reach convergence with the conjugate gradient method (our results demonstrate that this approach is the most efficient, see in the Results section) varied between 300 and 1600 for different small compounds. Therefore for further analysis, two protocols were assessed: one protocol employing two subsequent steps of 500 iterations and one with 1000 iterations. The minimized and initial structures were compared based on the RMSD values between the non-hydrogen atoms using the Superimpose option of the InsightII molecular modeling package [46]. We should notice that small molecule minimization results depend on initial conformations and this holds for any MM minimization engine. Problems with possible biases due to the starting conformation and exploration of other conformers can be circumvented by the use of our multiconformer generator Multiconf-DOCK [47] or of OMEGA prior to minimizing small molecules.

#### Energy minimization of small organic molecules using other force fields

Two MM minimization methods implemented in the program SYBYL were applied on the same set of small molecules in order to compare the minimization results obtained with AMMOS. The initial structures of the four small compounds, generated by OMEGA (see for details Compound collections), were optimized by two force fields: the Tripos force field (Tff) [36] and MMFF94s [48]. Tff minimization was performed with Gasteiger-Huckel charges, and MMFF94s, with MMFF charges. For both force fields, the following settings were used: distance-dependent dielectric function; non-bonded cutoff 8.0 Å; 0.02 kcal.mol^-1^.Å^-1 ^energy gradient convergence criterion; simplex initial optimization. For comparison, several runs were performed with 0.01 kcal.mol^-1^.Å^-1 ^convergence and no essential differences in the resulting geometries were recorded. Two gradient methods were experimented: Powell and conjugate gradient. Powell method [49] belongs to the conjugate gradient family of minimization methods. It is also more tolerant to inexact line searches. As a result, it is faster than the conjugate gradient method and is well-suited for a wide variety of problems [49]. The number of iterations was set to 5000 for both methods, in all cases however, the convergence was reached well below this number. Because the Powell and conjugate gradient results were quite similar, we report here only the data obtained with the Powell method. The minimized and initial structures were compared based on the RMSD values between the non-hydrogen atoms using the Match option in SYBYL. The calculations with SYBYL were performed on a Silicon Graphics Octane 2 (R12000) running under IRIX 6.5.

### Docking and scoring protocol

The pre-docked protein-ligand complexes subjected to energy minimization with AMMOS were generated via a multi-step docking-scoring protocol with DOCK6 [50]. DOCK6 accomplishes a sphere-matching algorithm to fit ligand atoms to spheres representing a negative image of the receptor-binding site. We used the program DMS [51] to compute the molecular surface of the receptor. The overlapping spheres within a radius of 4 Å were generated on the protein binding site surface using the program SPHGEN [52]. Sphere clusters within 6 Å to a reference ligand were retained for ER and 4 Å for NA (i.e., NA possesses a very open and flat binding site and it is important to limit the search on the active site area for this kind of target). The first docking step was carried out using rigid body-docking with DOCK6 applying the MS-DOCK protocol [47] over a compound collection of 37970 molecules (ADME/Tox filtered ChemBridge Diversity set) present in a multi-conformer state without initial minimization with AMMOS (see Compound collections). This stage assesses only shape complementarity and therefore, multi-conformer structures for the small compounds are needed in order to perform this fast "geometric" filtering step. For the positioning of the ligand in the binding site, we applied the faster manual match (see [50] and a maximum of 500 orientations). As mentioned above, in our calculations, the scores measured only the steric complementarity by use of the contact scoring function that counts the number of receptor-ligand contacts within a 4.5 Å distance from the ligand atoms. Each clash penalized the score by 30. The allowed bump overlaps were chosen to be 0.75 for NA and 0.50 for ER. These values were selected according to our previous observations [47]. We have seen that for large and flat cavities binding relatively small ligands like in the case of NA, a bump overlap of 0.75 improves the enrichment after a rigid docking procedure. On the contrary, when large ligands fill well the binding site, a bump overlap of 0.50 is preferable, a situation encountered with ER (see for details [47]).

Secondly, the retrieved non-minimized top ranked compounds (30–50 % of the library containing at least 60% of the actives) were directly re-docked using a flexible docking mode (i.e., flexibility from the ligand side) implemented in DOCK6 and employing the incremental built algorithm "anchor-first" [53] with our optimized parameters to better handle ligand flexibility [47]. We used a maximum of 1000 orientations for the anchor fragment. To speed-up the calculations, we set 50 configurations per cycle for the growth of the ligands. We applied 20 simplex minimization steps to each growth step. All docked molecules were ranked using the standard DOCK score involving soft van der Waals and distance-dependent electrostatic potentials. Finally for each ligand we saved up to 10 best scored conformers with a RMSD of 0.8 Å for subsequent minimization with AMMOS.

### Dataset preparation

#### Compound collections

Energy minimization with AMMOS and the Tff and MMFF94s force fields was carried out initially on 4 small molecules, taken from several X-ray protein-ligand complexes in the Protein Data Bank (PDB) [54], namely: **raloxifene **(an inhibitor of estrogen receptor, PDB code 1err); 4-(n-acetylamino)-3- [n-(2-ethylbutanoylamino)]benzoic acid (**FDI) **(an inhibitor of neuraminidase, PDB code 1b9s); **thymidine **(an inhibitor of thymidine kinase, PDB code 1kim); thieno [3,2-b]pyridine-2-sulfonic acid [2-oxo-1-(1h-pyrrolo[2,3-c]pyridin-2-ylmethyl)- pyrrolidin-3-yl]-amide (**PR2) **(an inhibitor of coagulation factor X, PDB code 1f0r).

To test the performance of AMMOS on a large number of small organic molecules we used the ChemBridge diversity set [55]. Our decoy library contained 37970 molecules after ADME/Tox filtering with the program Filter 1.0.2 [43]. We merged 20 known active compounds for ER and NA (with activities ranging from micromolar to nanomolar) and a number of rotatable bonds ranging from 4 to15) to the decoy collection. Some of the active compounds were taken from the PDB protein-ligand structures: 2 for ER (1err, 3ert; resolution 2.60 Å, 1.90 Å, respectively) and 10 for NA (1inf, 1inv, 1ivb, 1vcj, 1b9s, 1b9t, 1b9v, 1a4g, 1f8b, 2qwk; resolution 2.35–2.50 Å). When a ligand could not be extracted from the PDB, it was rebuilt from the literature [56]. All these active inhibitors were added to the decoy library, all in SMILES format. The resulting chemical library was transformed in single 3D conformer and saved in mol2 format using the program OMEGA 2.0 [43]. The multiconformer states were then generated by our program Multiconf-DOCK [47] applying an energy window of 25 kcal.mol^-1 ^and a diversity threshold of 1 Å RMSD. A maximum of 50 conformers were generated for each molecule. These values represent an appropriate balance between speed and accuracy according to the recent studies [47,57].

#### Protein targets

The performance of AMMOS for post-processing of the pre-docked protein-ligand complexes was validated on two protein targets. We selected ER with a closed and hydrophobic pocket and NA with an open and highly polar binding site. These two proteins present a binding site with very different degrees of burial (75.4% for ER and 30.5% for NA) and polarity (25% for ER and 65% for NA) (see for details [20]). We took the co-crystallized structures with best resolution among all retrieved protein-ligand complexes (PDB code 3ert, resolution 1.90 Å for ER and PDB code 1b9v, resolution 2.35 Å for NA). All bound water molecules and crystallized ligands were removed from the binding sites. Hydrogens were assigned using the program InsightII [46].

## Results

### Algorithm of AMMOS

**Figure 1:**
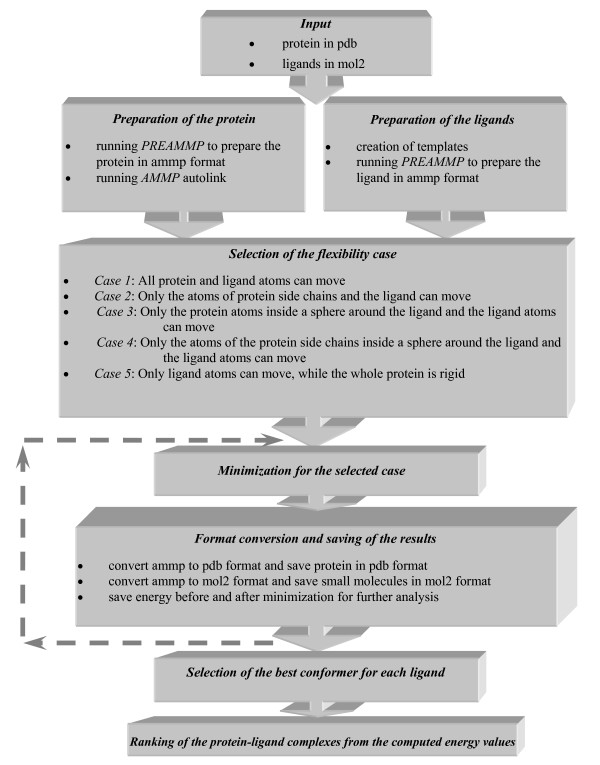
**Schematic diagram of the AMMOS procedure**. The arrows show the cycle of the automated procedure for a large number of ligands.

AMMOS drives a fully automatic procedure for minimization of protein-ligand complexes in a situation where the compounds are pre-docked in the binding site by any docking engine. AMMOS parameters are optimized such as to handle relatively large docked compound collections. Figure 1 illustrates all the different steps and required inputs, preparation, minimization of protein-ligand complexes with different degrees of protein flexibility, as well as final ranking of the ligands according to the minimized interaction energy between the ligands and the receptor. Firstly, AMMOS employs the program PREAMMP to convert the input protein and ligand files to AMMP format. Next, AMMP autolink is run to search for incomplete amino-acid residues and to finally link all the residues after corrections. AMMOS allows users to select one among five different solutions to handle protein-ligand complexes: from fully flexible minimization of the whole protein-ligand complex (*case *1) to a flexible ligand in a rigid receptor (*case *5) (see Fig. 1). In all situations, the ligand atoms are free to move. Our minimization step with AMMP applied on the protein-ligand complexes for the selected protein flexibility case involves 2×500 iterations with conjugate gradient optimization. The advances user can select any minimization method available in AMMP and specify the minimization parameters (i.e. number of iterations, convergence etc.). Finally, all the minimized conformers are scored by the AMMP minimized interaction protein-ligand energy [28] and re-ranked.

### Implementation of AMMOS

The package AMMOS consists of several programs developed in C and Python, and makes use of the open source programs AMMP and PREAMMP. One AMMOS routine (written in C) ensures the transformation of the input files (PDB for the protein and mol2 for the ligands) to a specific ammp format required by AMMP and at the end of the process generate reversely, the protein in PDB format and the small molecules in mol2 format. The automatization of the procedure described in Figure 1 for a large number of ligands in a single or multiple conformer state is accomplished via a Python script. Five different cases (scripts written in C) have been elaborated for the selection of the active/inactive atoms in the protein, while, in all situations, the ligand atoms are flexible: *case 1*: all atoms of the protein are active (a fully flexible minimization); *case 2*: all atoms of the protein side chains are active; *case 3*: all atoms of the protein inside a sphere around the ligand are active; *case 4*: all atoms of the protein side chains inside a sphere around the ligand are active; *case 5*: the whole protein is rigid. After processing of the whole pre-docked compound collection, the following results are saved in a subdirectory named *OUTPUT: *(i) the coordinates of all conformers after minimization, (ii) the coordinates of the flexible part of the protein after minimization, (iii) a file with warnings (if any), and (iv) the interaction energy between the protein and the ligands (external energy), the internal energies of the ligands or the protein, and total energy (including internal and external terms) before and after minimization. Finally a re-ranking step takes place based on the computed external energies of all minimized ligands. The method selects the best conformer among the multiple ligand conformers. The complete automatic procedure could be run for either sp4 or sp5 force field. Because sp5 was not explicitly available in AMMP, we created it using the extended and parameterized list of atom types and electronegativity values available in reference [39].

Another application of AMMOS is for the minimization of a large databank of chemical compounds in the absence of a protein. The procedure follows the scheme presented in Figure 1, and involves only the steps related to the small molecules, namely preparation and minimization of small molecules and final re-conversion to mol2 format.

All VLS and AMMOS calculations were carried out on one Mac Pro Quad-Core Xeon 3,0 GHz, 4Go RAM and on one Xeon 3.0 GHz Linux workstation, 1.5 GB RAM. The average time for the minimization on an Apple workstation was 0.3 sec for one small molecule and for one ligand pose in a protein was 24 sec for *case *5 (no protein flexibility is allowed), 44 sec for *case 3 *(all atoms of the protein inside the sphere around the ligand are active), and 5 min for *case 1 *(all protein and ligand atoms are active). Thus, execution times when a large number of ligands should be minimized with AMMOS can be very long. To speed-up the calculations, AMMOS allows jobs to be ran in parallel mode. The input mol2 file containing ligand molecules is divided into chunks of smaller numbers of molecules. During a parallel AMMOS run, each processor is assigned one chunk of molecules at a time. Final ranking of the complete databank containing the energy-minimized ligands is then accomplished after merging of the different chunks.

### Testing of AMMOS

#### I. Energy minimization of small organic molecules

Prior to test the automatic procedure for minimization of protein-ligand complexes, we first explored the ability of AMMOS to minimize small organic molecules starting from 3D structures generated by OMEGA. We compared several minimization methods available in AMMP (see *AMMP Molecular Mechanics *in the Methods section) and further compared the results obtained with AMMP with two other widely used force fields MMFF94s and Tff available in the SYBYL software.

##### Energy minimization of small molecules with AMMOS

The initial 3D structures of the four small molecules, raloxifene, FDI, thymidine and PR2 (see in Methods, Compound collections) generated by OMEGA were optimized using AMMOS with the force fields *sp4 *and *sp5*. Table 1 and Additional file 1 present the results for the four molecules and the five different applied optimization methods. For each method, the number of iterations needed to find the local minimum and the energy of the minimized structures are reported. As seen from Table 1, *sp4 *and *sp5 *provided very similar results in terms of optimized structural geometries. For PR2 and thymidine, lowest energies of the minimized structures with both *sp4 *and *sp5 *were achieved using the conjugate gradient method, followed by BFGS and GA. Conjugate gradient and GA gave the best results for raloxifene and FDI. For all tested molecules, steepest descent and simplex search led to minimized structures with higher energies. The above comparison suggests that conjugate gradient and GA produced lowest energies, however GA was much slower due to its stochastic nature. We also minimized structures containing important internal clashes in order to assess the robustness of the approach. After application of steepest descent the minimized structures remained insufficiently optimized, while conjugate gradient succeeded to overcome the clashes (results not shown). Although *sp5 *has been reported to be an improved version of *sp4 *[39], in our work *sp4 *minimized slightly better than *sp5 *in terms of energy differences between the initial and minimized structure, in particular for thymidine and raloxifene. Following the above observations, we chose conjugate gradient and *sp4 *as the minimization protocol for subsequent assessment of AMMOS.

**Table 1:** Comparison of AMMP optimization methods for small molecules minimized with sp4 and sp5 force fields

			**Optimization methods**									
			
**Compound**		**Initial Energy**	**Steepest descent**		**Conjugate gradient**		**BFGS**		**Simplex**		**GA**	
			
			N_iter_	E_min_	N_iter_	E_min_	N_iter_	E_min_	N_iter_	E_min_	N_iter_	E_min_
raloxifene	sp4	121.08	20	86.72	1600	66.71	1500	75.89	80	102.24	300	69.97
	sp5	117.41	20	88.46	1000	71.65	1200	77.62	100	107.03	300	70.89
	RMSD			0.01		1.23		0.05		0.14		0.42

FDI	sp4	119.61	30	74.02	200	66.14	1200	64.38	10	268.07	300	48.95
	sp5	116.89	30	75.48	300	67.94	1000	67.96	10	283.08	200	51.83
	RMSD			0.01		0.12		0.02		0.14		1.14

thymidine	sp4	99.64	30	64.82	400	60.52	500	61.17	20	114.81	300	61.40
	sp5	95.78	20	68.49	200	64.29	1000	64.57	10	99.78	200	65.85
	RMSD			0.01		0.09		0.03		0.06		0.63

PR2	sp4	156.55	50	-5.22	300	-14.57	700	-13.58	10	78.01	200	-5.77
	sp5	155.04	50	1.11	300	-13.01	600	-11.77	20	72.18	200	-12.73
	RMSD			0.07		0.02		0.01		0.02		0.61

The number of iterations needed to reach convergence (Niter) and the minimized energy (Emin in kcal/mol) and the RMSD values (in Å) between the optimized structures with sp4 and sp5 force fields are given.

As seen from Table 1, the required number of iterations for convergence (0.02 kcal.mol^-1^.Å^-1 ^energy gradient convergence criterion) with *conjugate gradient *varies between 200 and 1600 for different molecules. The four molecules show different structural properties with various numbers of rotatable bonds: 7 (raloxifene), 6 (FDI), 5 (PR2) and 2 (thymidine). Raloxifene and PR2 are larger than the other two molecules with molecular weights of 473 and 453, respectively. One can see from the figure reported in the Additional File 1 that after 400 iterations with *conjugate gradient *the energy gradients of all molecules are very close to the convergence criterion. Our additional tests confirmed that using 1000 or 2× 500 iterations are an acceptable compromise between geometric optimization and speed. Thus, we accepted 2×500 iterations as an appropriate number of steps for the automatic AMMOS procedure aiming at minimizing thousands of small organic molecules or of protein-ligand complexes (i.e., users can change these values if needed).

Further, we examined 4 different potential functions for non-bonded interactions as implemented and named in AMMP: 1) *nonbon *– involving point atom electrostatics; 2) *screen *– 1s distributed charge electrostatics; 3) *debye *– Debye screened potentials and 4) *shadow *– 4-D non-bonded for embedding. Because compared to the *nonbon *potential, no significant differences were found with the *debye*, *screen *and *shadow *modes and since they considerably increase the lengths of the calculations, we selected the *nonbon *potential for further evaluation of AMMOS (users can employ the *debye *or *shadow *options scripts to account for screening solvent effects).

##### Energy minimization of small molecules with other force fields

The results achieved after application of AMMOS minimization with conjugate gradient with 2× 500 iterations and *sp4 *(this protocol is employed by the automatic procedure for a large number of molecules) were compared with two other force fields – MMFF94s and Tff applied on the same four small molecules starting from 3D initial structures generated by OMEGA. Similar results in terms of energies and structural geometries were obtained with Powell and conjugate gradient methods when one of the MMFF94s and Tff force fields were used. Therefore only the results with the method of Powell for both force fields MMFF94s and Tff are reported here. Table 2 shows the energies of the initial (E_ini_) and minimized (E_min_) structures, obtained with AMMOS, MMFF94s and Tff, as well as the energy differences (ΔE) between the initial and minimized structures. Although energy values obtained by different force fields are not directly comparable, the differences between the energies of the initial and minimized conformations can be indicative of the optimization performance to search for the local minimum. As seen from Table 2, generally there is a correlation between ΔE values in AMMOS and the other force fields, thus confirming that AMMOS is able to remove ligand energy strains.

**Table 2:** Energy minimization with the AMMP sp4, MMFF94s and Tff force fields

**Compound**	**AMMOS, sp4**			**MMFF94s**			**Tff**		
	
	E_ini_	E_min_	ΔE	E_ini_	E_min_	ΔE	E_ini_	E_min_	ΔE
raloxifene	121.08	69.82	51.26	100.49	86.18	14.31	61.27	16.42	44.85

FDI	119.61	66.14	53.47	32.41	23.81	8.60	59.79	-1.03	60.82

thymidine	99.64	60.52	39.12	-40.41	-58.83	18.42	22.7	-1.58	24.28

PR2	156.55	-14.57	171.12	19.59	-43.31	62.90	146.95	10.27	136.68

Energies of the initial (Eini) and optimized (Emin) structures are given in kcal/mol.

**Figure 2:**
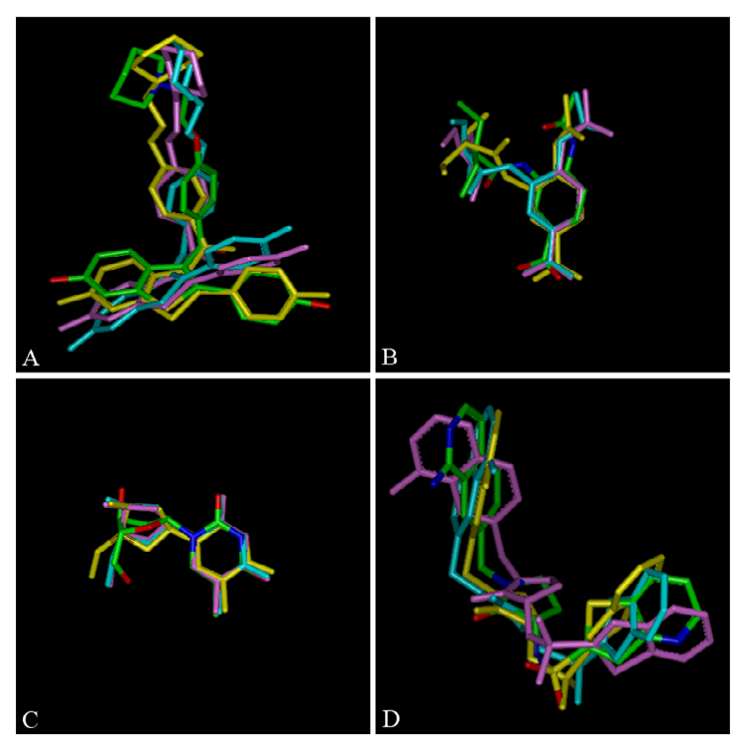
**Structural refinement of four small molecules with AMMOS (magenta), MMFF94s (cyan) and Tff (yellow)**. The minimized structures are superimposed on the corresponding X-ray structures (all atom colored). Raloxifene (A), FDI (B), thymidine (C) and PR2 (D).

Table 3A reports the RMSD values (all heavy atoms) between the initial and optimized structures for the four small molecules with the three force fields. When comparing the RMSD values between the optimized and initial structures similar results are observed between AMMOS and MMFF94s, as could also be seen in Figure 2 which represents the superimposition (all heavy atoms) of the structures minimized by AMMP, MMFF94s and Tff. In conclusion, the results obtained after minimization with AMMOS are close to those obtained by other force fields. A widely accepted quality measure for the predicted conformations of small molecules is the RMSD from structures extracted from crystal protein-ligand complexes [57,58] despite the fact that the experimental structure does not necessarily correspond to the lowest-energy conformation [58-60]. The RMSD values between our minimized and X-ray structures are given in Table 3B. The relatively low RMSD values between the optimized and X-ray structures suggest that AMMOS produces reasonable and reliable small compound structural geometries.

**Table 3:** RMSD values (in Å) obtained after matching the optimized and initial structures (A) and the optimized and X-ray structures (B)

**A**	**AMMOS**	**MMFF94s**	**Tff**
raloxifene	0.80	0.84	1.34
FDI	0.14	0.49	1.13
thymidine	0.15	0.26	0.51
PR2	0.40	1.13	1.23

**B**	**AMMOS**	**MMFF94s**	**Tff**

raloxifene	2.12	2.39	1.51
FDI	1.88	1.90	1.52
thymidine	1.03	1.01	1.17
PR2	1.69	1.68	1.57

The minimization with AMMOS is performed with 2×500 iterations and sp4 force field.

##### Energy minimizations of a large chemical library with AMMOS

**Figure 3:**
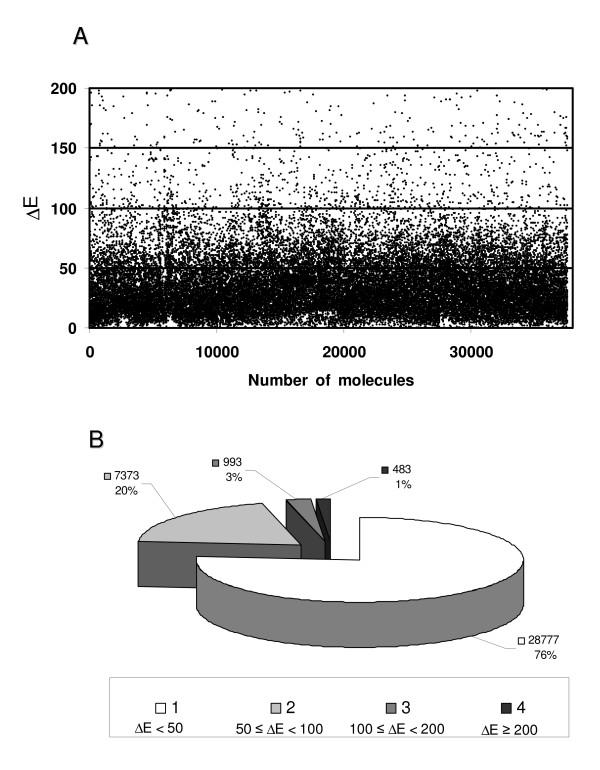
**Energy minimization results for a chemical library of 37970 small organic molecules**. A. Scatter-plot of the energy differences ΔE in kcal/mol between the AMMOS minimized and initial structures; B. Pieplot of the ΔE distribution.

The AMMOS procedure for minimization of small compounds was applied to a chemical library of 37970 single conformer molecules (see Methods, Compound collections). Figure 3A shows that the differences (ΔE) between the energies of the AMMOS minimized and initial structures generated by OMEGA can be up to around 200 kcal.mol^-1^. Figure 3B shows in a more illustrative way the ΔE distribution in the compounds databank: for 76% of the molecules ΔE is below 50 kcal/mol, and only 4% of the compounds show ΔE values > 100 kcal/mol. It should be noted, that AMMOS succeeded to minimize molecules with very high initial energies. The AMMOS minimization improves the geometries thus making the optimized structures more appropriate for subsequent docking and scoring. It is interesting to note that we did not succeed to optimize several structures presenting severe internal clashes and thus very high energies with the other minimization protocols like, for instance, steepest descend, at least as implemented in the AMMP package. This is of interest since, when the derivatives of the energy function are high, it is usually accepted that steepest descents is the most appropriate minimizer. Overall, these results and tests demonstrate the efficiency of AMMOS in the structural refinement of a compound collection.

#### II. Energy minimization of protein-ligand complexes with AMMOS

##### Generation of docked protein-ligand complexes

Prior to the minimization step carried out with the AMMOS procedure, the protein-ligand complexes were generated using a two-step docking protocol (see Methods). The decoy library contains 37,970 drug-like molecules and two protein targets were used for the VLS experiments. The 37,970 compounds were docked/scored on each protein target, NA and ER, employing first rigid body docking performed with our MS-DOCK protocol [47] and next flexible docking (from the ligand side) with DOCK6. The top ~30% best scored molecules were selected after the rigid docking step and 6 known actives for ER out of 10 were found. In the case of NA, we decided to select about 50% of the best-scored molecules after the rigid docking, and among these, 6 actives out of 10 were present. In our previous study [47], we demonstrated that for large ligands filling well the binding pocket (which is the case of ER) selecting the top 30% of the database after shape-complementarity filtering by rigid docking gives a sufficient enrichment for a subsequent flexible docking step. When relatively small ligands bind to a very open and flat pocket (which is the case of NA), 50% of the top ranked ligands after the first rigid docking step should be selected for the subsequent flexible docking step. After flexible docking, we disposed of two target-specific compound libraries, each containing 12,000 compounds (52,345 conformers) for ER and 22,000 compounds (70,120 conformers) for NA. These two collections containing pre-docked ligands in a multi-conformer state were subsequently subjected to the AMMOS minimization protocol.

##### Enrichment studies using AMMOS

We next investigated the impact of the minimization protocol and rescoring with AMMOS on enrichment. The AMMOS runs were performed on ER and NA for the five developed cases (see Figure 1 and the Methods for details). *Case 1 *and *2 *(full flexible receptor during the minimization) were carried out only for the real actives to reduce the calculation time. Calculations within the framework of *cases 3*, *4 *and *5*, with a partial flexibility of the protein within a 6Å sphere around the ligand (this is a user defined parameter), were performed for the entire pre-docked chemical library. As mentioned above, we used conjugate gradient with the *sp4 *force field. We decided to apply two cycles of 500 iterations in order to have an acceptable compromise between level of minimization and calculation time.

**Figure 4:**
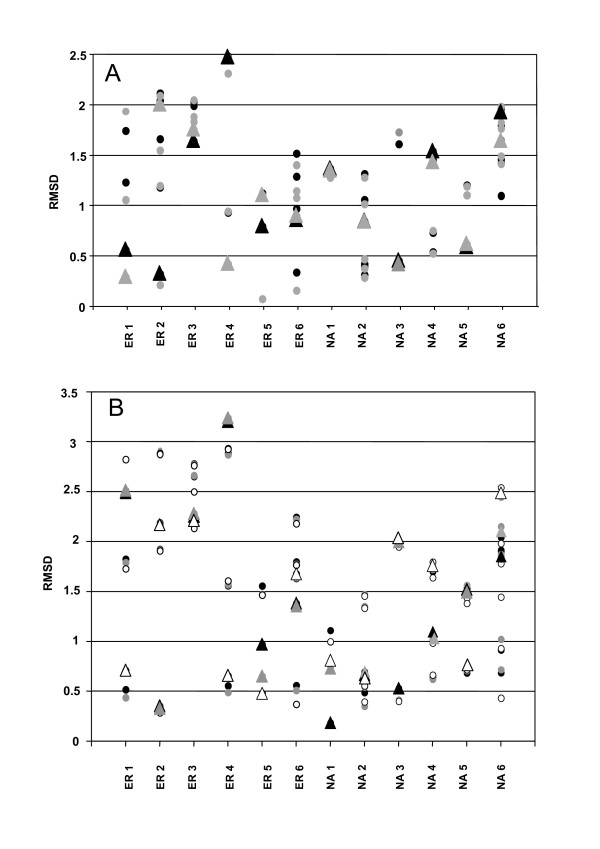
**RMSD (Å) between the AMMOS minimized and X-ray structures for the inhibitors retrieved for the ER and NA targets**. A. Each point represents a single conformer minimized with *case 1 *(black), *case 2 *(grey); Triangles refer to the conformers with lowest energy after AMMOS minimization; B. All conformers are represented by points, with respectively black for *case 3*, grey for *case 4 *and white for *case 5*. Triangles refer to the conformers with lowest energy after AMMOS minimization.

Figure 4 illustrates the RMSD between the X-ray and AMMOS minimized structures (applying all *cases *1–5) for 6 ER and NA inhibitors retrieved after DOCK6 flexible docking and saved in a multi-conformer state (up to 10 poses per ligand). Although ligands bound to proteins are not always in the lowest-energy conformation [59], several recent studies suggested that the bioactive ligand structure should not deviate substantially from a low-energy state [61]. Thus, the lowest-energy conformers are also shown in Figure 4. As seen in this figure, for all shown inhibitors but ER3, the best fitting conformers, with all *cases 1–5*, show RMSD values with the experimental structure below 1.5 Å, highlighting further the performance of AMMOS minimization [12]. Slightly better results were obtained for ER when the whole receptor was involved in the minimization (Fig. 4A) compared to the data in which the binding pocket and/or the ligand alone were taken into account (Fig. 4B). In the case of NA, important differences depending on the involved receptor level in the minimization were not observed.

**Figure 5:**
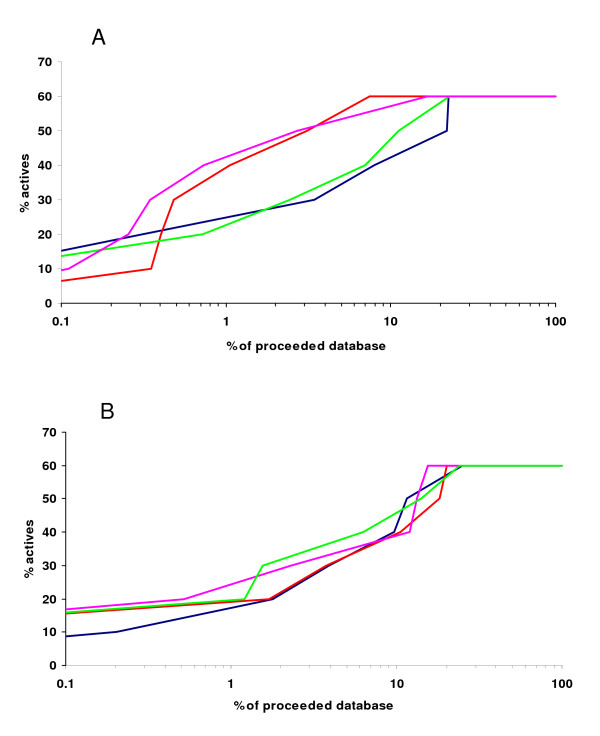
**Enrichment graphs for ER (A) and NA (B) inhibitors after AMMOS minimization and rescoring**. The y-axis is the % of retrieved actives versus the percentage of the database screened (x-axis): enrichment results after flexible docking step (blue); enrichment results after re-scoring employing AMMOS minimization: *case 3 *(red), all protein atoms inside a sphere around the ligand can move; *case 4 *(magenta), all side chain protein atoms inside a sphere around the ligand can move; *case 5 *(green), the whole protein is rigid.

Further we validated the importance of using AMMOS as a final step of a hierarchical VLS protocol. We used a two-stage VLS protocol and a compound collection of 37,970 compounds to generate and select protein-ligand complexes with satisfactory shape complementarity, thus reducing the number of protein-ligand complexes to be minimized. The final selected pre-docked protein-ligand complexes for both protein targets containing 60% of the real active compounds were subjected to energy minimization with AMMOS. Figure 5 shows the enrichment graphs for the two targets before and after application of the AMMOS minimization protocol. Enrichments for the ER inhibitors (Figure 5A) when employing *cases 3 *(red) and *4 *(magenta) are better than with the *case 5 *(green). In both *cases 3 *and *4*, AMMOS retrieved 50% of the inhibitors in the top 3% (1200 compounds) of the entire database.

NA is known as a "difficult" protein target because its binding pocket is open and flat and thus challenging for docking-scoring methods. Despite these difficulties, results obtained after the three-minimization cases with AMMOS (Figure 5B) are reasonable. Again, 50% of the inhibitors are found in less than 10% of the proceeded database. It is interesting to note that when the protein was kept rigid (*case 5*, green), slightly better results for NA were achieved after AMMOS minimization. The above results suggest that AMMOS performs well and its use can help to improve enrichment in multi-step SBVLS protocols.

## Discussion

Optimization of existing SBVLS tools and development of open source packages are of crucial importance to assist drug discovery projects. In this work, we developed a new software tool named AMMOS that refines the 3D structure of compounds present in a compound collection, optimizes the binding poses of docked ligands through MM minimization, and rescores these protein-ligand complexes. The open source package AMMOS wraps and enhances the functionality of the MM simulation program AMMP, facilitating its use for *in silico *screening. Numerous algorithms and protocols can be used to perform SBVLS experiments but in most cases, the receptor structure remains rigid during the docking/scoring procedure. Similarly, compounds obtained from chemical vendors are in 2D and their 3D structures have to be generated. Several tools can perform this task but rarely the compound 3D structures are refined prior to docking while it is known that this can be critical for positioning and obviously scoring [19]. Further and in order to speed-up the SBVLS process, hierarchical protocols have been developed. These ones can include a relatively crude initial shape complementarity filtering step (e.g., rigid docking with compounds present in multi-conformer states) followed by flexible docking with flexibility allowed only for the ligands. In several situations, it could be beneficial for users to have a tool allowing for partial to fully flexible energy minimization of the protein-ligand complexes, either to help ranking or to optimize the docked poses prior to selecting a list of molecules for experimental assay. AMMOS can be applied in these situations as illustrated below.

### Structural refinement of the compound collections with AMMOS

Some recent reports suggest that it could be beneficial to minimize the 3D structure of the small molecules before performing docking/scoring computation [18,19,59]. The question is indeed complex because it is widely accepted that ligands do not necessarily adopt their lowest potential energy conformations when bound to a protein. In fact, ligand energies of bioactive conformations have been estimated to be around 5 kcal/mol above the lowest conformation energy of the same compound in solution [59]. Further, a recent study carried out on 100 protein-bound ligand crystal structures shows that the ligand conformations are nearly identical to their local minimum conformations obtained from normal mode analysis-monitored energy minimization [18]. All these studies demonstrate that the ligands bind to proteins in a relatively low energy state, suggesting that highly strained energy input ligand conformations should not be appropriate for docking procedure, keeping in mind that many docking algorithms alter the pose of their input ligands only through translation, rotation, and some dihedral angle variations at rotatable bonds. Minimizing compound collections prior docking experiments was demonstrated to improve both docking and scoring with Surflex [19]. Our preliminary tests involving docking (with DOCK6) of ER inhibitors initially minimized with AMMOS (see Additional File 2) indeed suggest that minimizing small molecules prior to docking can improve at least the docking accuracy. Thus, as seen in the result section, AMMOS is able to automatically energy minimize a large compound collection. The resulting compounds have less strain energy but remain close from experimental structures and display similar structural features as compounds energy minimized with two other well-known force fields.

### Refinement of protein-ligand complexes and re-scoring with AMMOS

It is important to note that, at present, it is generally accepted that MM methods are not well-suited for a large scale VLS computations because they are slowing down the calculations and since it is unclear whether or not energy minimization with current force fields could help in the ranking process [62] (i.e., does MM minimization optimize the poses, does computed interaction energy after energy minimization is beneficial to ranking). Some of these questions start to be addressed as for instance several recent studies suggest that post-docking energy minimization can significantly improve enrichment for some protein targets [24-26]. It is expected that rescoring after MM minimization cannot help when the ligand is wrongly positioned in the binding cavity, however, when the pose generated by a docking engine is near the experimental conformation, energy minimization seems to be able to optimize locally the structure of the complexes, and in this case, the computed energy values (based on traditional force fields or via other scoring approaches) of the minimized structures can be effective at improving ranking and enrichment [24].

In the present study, we provide users with the AMMOS package that refines the structure of pre-docked ligand-protein complexes. To illustrate our method, we applied a two-step docking-scoring VLS protocol (i.e., rigid body docking with MS-DOCK and subsequent ligand flexible docking with DOCK6) on two protein targets, ER and NA. Then, we minimized the 10 best poses obtained with DOCK6 with AMMOS and re-scored each pose. Enrichment curves were obtained with different levels of allowed receptor energy minimization (Figure 5). As seen, employing fully flexible minimization in the binding site can considerably improve enrichment as it was observed in the case of ER with 50% of the inhibitors retrieved in the top 3% of the entire database. Thus, users can select different protocols depending on the projects and needs (e.g., consider small local flexibility in the binding site area). In terms of efficiency, enrichments obtained by AMMOS are similar to those seen by other tools such as PLOP [26]. Indeed, after AMMOS rescoring, we found 40% of the NA inhibitors and 60% of the ER active compounds in the top 5% of the compound libraries (2000 compounds).

Although the post-docking MM minimization of docked poses can improve the binding modes and the scoring in VLS, it has been also observed that relaxing the protein-ligand complexes for some proteins can worsen the final enrichment although the binding modes are improved. For instance the MM scoring method PLOP [25] involving a generalized Born solvent model has been shown to improve the overall enrichment only for five out of ten tested protein targets. In average, for these ten targets, better enrichment factors were obtained in the top 0.5% of the proceeded database, however only 10–30% of the inhibitors were retrieved in this limited range. Further similar rescoring via the molecular mechanics-generalized Born surface area (MM-GBSA) method [63] with relaxation of the protein-ligand complexes by MM minimization with PLOS or short molecular dynamics (MD) with AMBER-DOCK on three simple ligand binding cavities helped to discriminate between several active inhibitors and experimentally proven decoy molecules. Here again, the obtained enrichment factors were lower than those found by docking alone before relaxation and rescoring with MM-GBSA. In that study the authors proposed that ligands too large to be accommodated after docking could indeed fit after relaxation of the binding site. In addition, some imperfections in the balance between polar interactions and solvation penalties can be present in the MM-GBSA methodology.

Along the same line of reasoning, in our study and in the case of ER where large ligands fill well the binding site (with the ratio Volume of the ligand/Volume of the pocket of 0.95) allowing receptor relaxation with AMMOS (*case *3 and *case *4) led to considerable improvement of the enrichment factors (see Fig. 5A). On the other hand, we did not obtain better enrichment after AMMOS in the case of NA (Fig. 5B). Also, our additional tests with AMMOS applied on a third protein target, thymidine kinase, did not improve the final enrichment, particularly with *cases *3 and 4 when local receptor flexibility was allowed (data not shown). It seems, that for some binding pockets and ligands, allowing post-docking relaxation of the protein-ligand complexes may worsen the enrichment, thereby increasing the number of false positives, as we observed not only in our investigation of NA and thymidine kinase but also in other studies [25,63]. For NA and thymidine kinase, the ligands do not fill well the binding sites (with a Volume of the ligand/Volume of the pocket ratio of 0.44 and 0.45, respectively). Thus, it can be speculated that for large pockets accepting relatively small ligands, authorizing flexibility around the binding site could damage the final enrichment.

In terms of speed, AMMOS can perform full atom minimization of the binding site area in about ~30–45 sec/ligand conformer with the default parameters. The protocol provided offer a reasonable compromise between speed and accuracy but the users can reduce the number of iterations and speed-up the calculations, depending on the aims of the projects. In addition, multiple poses optimized with AMMOS can be subsequently re-ranked by employing other scoring functions or consensus scoring [2,16].

## Conclusion

An efficient MM minimization engine called AMMOS that makes use of the AMMP simulation package has been presented. The fully automatic open-source package AMMOS can perform energy minimization of a large compound collection prior to docking and can also refine pre-docked ligands in the context of a protein structure. AMMOS executes efficient and fast minimization procedures and can thus be employed over a large number of protein-ligand complexes. The users can select the energy minimization protocol depending on the projects and, for instance, fix the protein atoms or allow full flexible minimization of both, the ligand and the receptor. Several initial tests were carried out in order to assess AMMOS. We applied our package on two protein targets, ER and NA, which have very different binding site properties. Results shows that AMMOS performs well on large pre-docked compound collections, retrieving most of the active compounds in the top 3% to 5% of the entire database. Although MS-DOCK and DOCK6 were used in this work, users can apply other docking programs to dock a compound library.

The present version of AMMOS does not take into consideration possible solvation effects, yet, a few water molecules could be treated by the minimization procedure. However, in such a situation, we do not provide a fully automatic procedure and the users should prepare the proper input files. Automated protocol will be implemented in a future release of AMMOS to treat solvation and water molecules. The full AMMOS package is freely available and should be valuable to research groups involved in drug discovery and chemical biology projects.

## Availability and requirements

AMMOS is written in C and python and runs on Linux platforms. It is also operational on MacOSX system. Detailed installation instructions are provided in the software package. The source code of AMMOS is freely available under the terms and conditions of the GNU Public License from .

## Authors' contributions

TP wrote the code in C for the AMMOS project, tested the program and drafted the first version of the manuscript. DL wrote and tested the Python code for the automated procedure. IP ran the molecular mechanics computations with the SYBYL software. BOV and MAM conceived the AMMOS tool. MAM designed and managed the project. IP, BOV and MAM took an active part in manuscript writing. All authors read and approved the final manuscript.

## Supplementary Material

Additional file 1 **Energy minimization of small molecules with sp4 force field and different AMMP optimization methods.** The minimized energies by the Steepest descent, Conjugate gradient, BFGS, Simplex and a genetic algorithms are plotted against the number of iterations. Click here for file

Additional file 2 **Docking poses of estrogen receptor inhibitors after flexible docking with DOCK6.** The docking poses of six inhibitors minimized with AMMOS prior to docking as well as the docking poses of the inhibitors not minimized prior to docking are shown. Click here for file
