# Supplementary material for: AMMOS: Automated Molecular Mechanics Optimization tool for in silico Screening
Source: BMC Bioinformatics. 2008 Oct 16;9:438. doi: 10.1186/1471-2105-9-438 (PMC2588602; doi:10.1186/1471-2105-9-438)
Supplement: Additional file 1 — Energy minimization of small molecules with sp4 force field and different AMMP optimization methods. The minimized energies by the Steepest descent, Conjugate gradient, BFGS, Simplex and a genetic algorithms are plotted against the number of iterations. [file 1471-2105-9-438-S1.pdf]

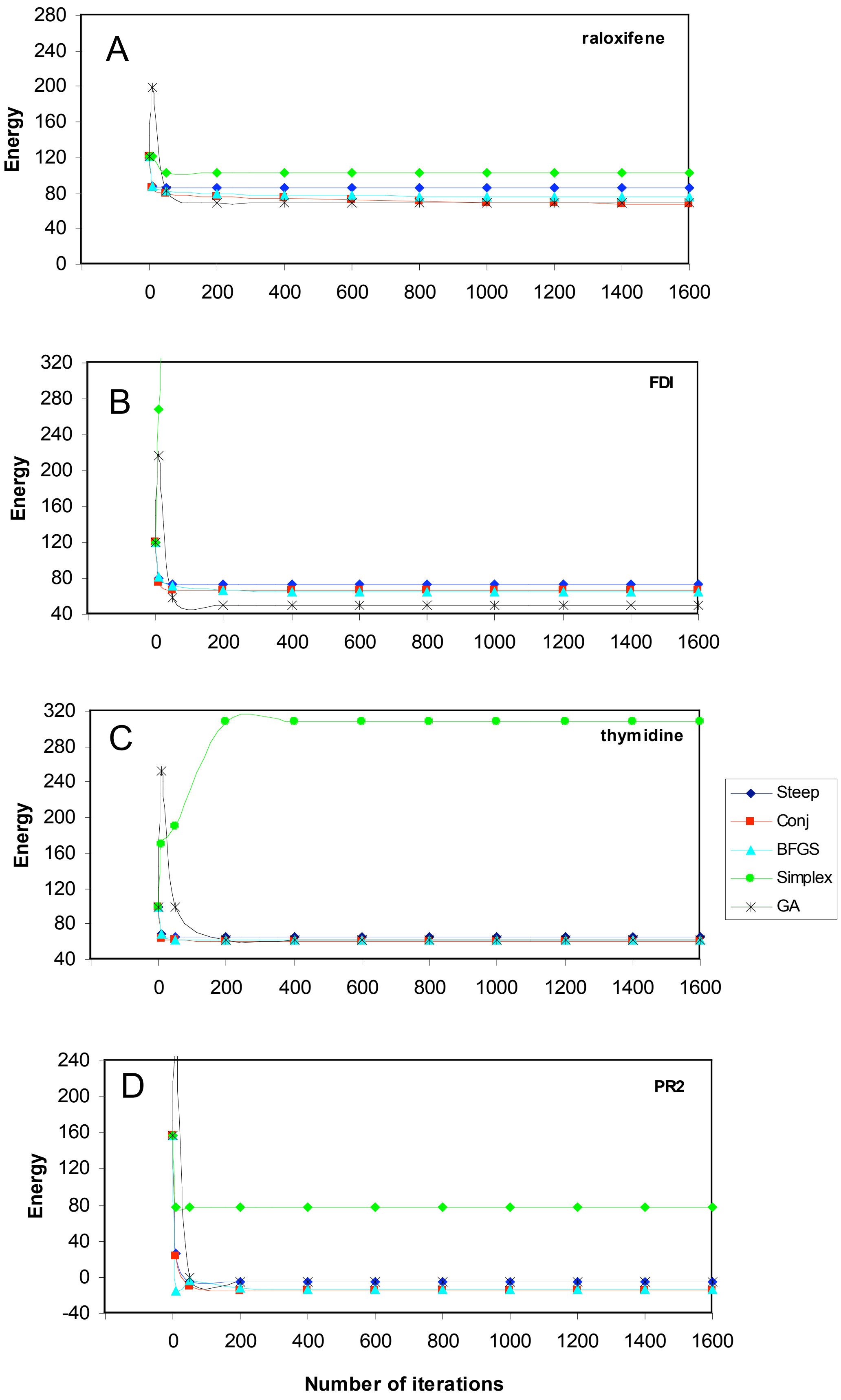

Figure 1

Energy minimization of small molecules with sp4 force field and AMMP optimization methods: Steepest descent (Steep), Conjugate gradient (Conj), BFGS, Simplex and a genetic algorithm (GA). The minimized energies (**Energy** in kcal/mol) are plotted against the **Number of iterations**. A: raloxifen; B: 4-(n-acetylamino)-3-[n-(2-ethylbutanoylamino)]benzoic acid (FDI); C: thymidine; D: thieno[3,2-b]pyridine-2-sulfonic acid [2-oxo-1-(1h-pyrrolo[2,3-c]pyridin-2-ylmethyl)-pyrrolidin-3-yl]-amide (PR2).
