# Supplementary material for: AMMOS: Automated Molecular Mechanics Optimization tool for in silico Screening
Source: BMC Bioinformatics. 2008 Oct 16;9:438. doi: 10.1186/1471-2105-9-438 (PMC2588602; doi:10.1186/1471-2105-9-438)
Supplement: Additional file 2 — Docking poses of estrogen receptor inhibitors after flexible docking with DOCK6. The docking poses of six inhibitors minimized with AMMOS prior to docking as well as the docking poses of the inhibitors not minimized prior to docking are shown. [file 1471-2105-9-438-S2.pdf]

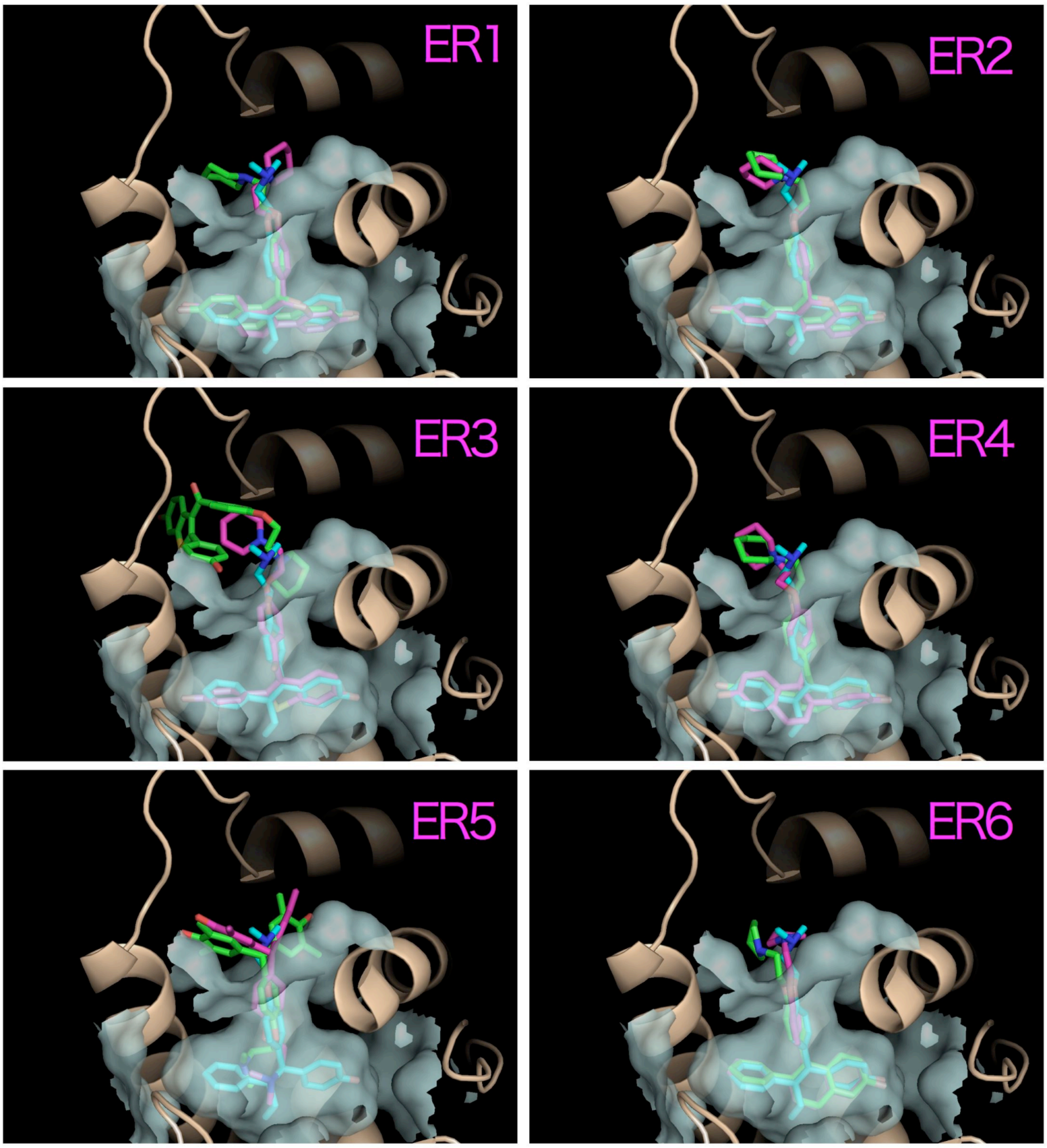

Figure 1

Docking poses of estrogen receptor (ER) inhibitors after flexible docking with DOCK6. The binding site of ER (pdb code 3ert) is represented by a molecular surface and ribbons. Within each panel the co-crystallized 4-hydroxytamoxifen (sticks with cyan carbon atoms) and two docking poses for one of ER inhibitors are shown. The docking poses of the inhibitors minimized with AMMOS prior to docking are represented as sticks with magenta carbon atoms. The docking poses of the non-minimized inhibitors prior to docking are represented as sticks with green carbon atoms. The figure is rendered with the program PyMOL.
